# Anaphylaxis and loss of wheat tolerance after two weeks of wheat deprivation

**DOI:** 10.1186/s13223-026-01023-3

**Published:** 2026-03-10

**Authors:** Hannah Martin, Edmond S. Chan, Raymond Mak, Angela Maccan

**Affiliations:** 1 Faculty of Medicine, University of British Columbia, 2350 Health Sciences Mall, Vancouver, BC Canada; 2 Division of Allergy, Department of Pediatrics, BC Children’s Hospital, University of British Columbia, Vancouver, BC Canada

**Keywords:** Wheat allergy, Allergen deprivation, Allergen reintroduction, Allergy prevention, Oral immunotherapy

## Abstract

**Background:**

The best practices for childhood allergy prevention are continually evolving. We do not know the optimal frequency for continued exposure of common allergenic foods after the initial introduction into a child’s diet. Home-based oral immunotherapy is a promising treatment avenue for childhood food allergies.

**Case presentation:**

We describe the case of a child who was tolerating wheat and underwent a 2-week unintentional deprivation period wherein they were not exposed to any wheat. After this period, they experienced an anaphylactic reaction to wheat upon re-introduction. The child had a history of other allergies for which they were receiving oral immunotherapy and they now are receiving oral immunotherapy for wheat as well.

**Conclusions:**

Our case is unique as, to the best of our knowledge, there has yet to be a case published wherein a deprivation period as short as 2 weeks has resulted in an anaphylactic reaction, despite previous tolerance of the allergen. Clearer labelling of gluten free versus gluten-containing products as confusion regarding labelling contributed to the unintentional deprivation period is needed. Families should also be counselled on thorough food product label reading. Finally, further research is warranted regarding the optimal frequency of potential allergen dietary reintroduction.

## Background

To prevent allergy development, common allergens should be introduced to infants around the age of 4–6 months [1]. After introduction, the optimal frequency of continued ingestion is unknown [2]. Home-based oral immunotherapy, in which parents are taught how to introduce small amounts of the allergen into the child’s diet at home with scheduled dosage increases, is an emerging treatment option [3, 4].

IgE-mediated wheat allergy affects approximately 0.2% of children in Canada [5]. The development of IgE-mediated wheat allergy after prolonged wheat tolerance is mainly described in the literature in patients with celiac disease, previously eating wheat regularly, but developing wheat allergy after a gluten-free diet [6]. Wong et al. wrote of a Canadian child with celiac disease who developed comorbid IgE-mediated wheat allergy [7].

## Case presentation

Our case covers a 22-month-old male who developed an anaphylactic reaction suspicious for wheat allergy following two weeks of unintended deprivation. The patient’s family was known to our clinic, the BC Children’s Hospital Pediatric Allergy Clinic, as his older brother was receiving tree nut oral immunotherapy. The parents had been counselled regarding early food introduction and regular exposures to allergenic foods, but at age 10–15 months, the patient was identified as allergic to peanut, egg, soy and almond and began receiving virtually supervised OIT for these allergens at 16 months. Table 1 provides results of skin prick testing at 11 months, with positive tests to egg white, egg yolk, peanut and almond. Table 2 summarizes serum specific IgE (sIgE) values to each food tested.

At 18 months, the mother accidentally bought a gluten-free version of his regular wheat-containing cereal due to similarities in packaging, causing an unintended and unrecognised 2-week deprivation without wheat consumption after which his mother gave him bites of a home-made wheat-containing muffin, previously tolerated. He immediately developed hives and coughing. His mother administered children’s loratadine and drove him to the emergency department where his symptoms resolved within 30 min. The following week he was given a grilled cheese sandwich (containing wheat bread), the only wheat exposure since his reaction to the muffin, thereupon developing cough and generalized hives. He was administered loratadine and became fussy. His coughing quickly worsened, and breathing difficulty resulted in his mother administering epinephrine, with near-immediate reduction in his symptoms. Subsequent hospital observation indicated no further treatment was required. Prior to 18 months, he tolerated wheat regularly for approximately one year (wheat puffs started at 6 months, together with muffins and grilled cheese sandwiches several times/week). During the 2-week deprivation period he was coincidentally not given these other wheat sources.

Following these two episodes of anaphylaxis, the patient was again seen in our clinic. At 20 months, SPT to fresh wheat was positive at 12 × 15 mm with wheat sIgE of 11.6 kU/L, confirming new-onset wheat allergy. At this time, he had nearly attained maintenance dosing for peanut, egg, soy and almond after almost 4 months of OIT, with significant decreases in the associated skin prick test sizes. The patient’s mother was experienced with the process of OIT for the first 4 allergens, after participating in our hybrid program of 5 virtually supervised build-up doses followed by home-based escalations for remaining doses. Therefore, she was comfortable adding wheat using an entirely home-based OIT buildup. At a follow-up visit at 22 months, the patient had reached maintenance dosing to peanut, egg, soy and almond, and was progressing well with home-based wheat OIT buildup with only mild, intermittent skin symptoms.

**Table 1:** Skin prick test results of the patient at 11 and 20 months old

	11 months old	20 months old
Peanut	13 × 6 mm	8 × 10 mm
Egg white	15 × 5 mm	10 × 8 mm
Egg yolk	10 × 5 mm	6 × 9 mm
Soy	Not tested	8 × 7 mm
Almond	5 × 3 mm	3 × 3 mm
Wheat	Not tested*	12 × 15 mm

*The patient was regularly tolerating wheat at this point in time.

**Table 2:** IgE results of the patient at 8, 11 and 20 months old. Units are kU/L

	8 months old	11 months old	20 months old
Peanut	26.70	18.9	25.20
Egg White	Not tested	19.3	9.05
Egg Yolk	Not tested	4.63	1.25
Soy	Not tested	7.67	6.70
Almond	Not tested	4.35	2.48
Wheat	Not tested	Not tested	11.60

### Discussions and conclusions

Prevention of childhood food allergies is challenging; caregivers are often busy and struggle to consistently feed potential allergens after first exposure, and children may reject foods unexpectedly. Ponda and Sicherer published a case report in which ingestion of Play-Doh caused an allergic reaction in a wheat-allergic child [8]. The Play-Doh website had a warning that their product contained wheat, but there was no warning on the product packaging [8]. An already-sensitized child thus consumed a product without labelling of the allergen [8]. In our case, a child consuming a specific cereal product unknowingly discontinued wheat exposure because the brand’s packaging was similar in appearance for wheat-containing and gluten-free varieties. To ensure parents provide the intended allergenic ingredient frequently, anticipatory counselling could be considered on careful label reading to ensure regular exposure, much in the same way that we may conversely counsel families to read labels carefully to avoid known allergens.

Allergy prevention becomes more difficult when as little as two weeks of deprivation can result in loss of tolerance and anaphylactic food allergy. A Canadian study suggested that once a month peanut consumption in younger siblings of peanut-allergic children yielded low risk of peanut sensitization, indicating infrequent exposures in the younger sibling resulted in easier management of the older sibling’s avoidance [9]. However, that study followed children with a higher median age (23 months), contrasting with most literature on primary prevention (including randomized controlled trials) of infants reliant on more frequent ingestion of introduced allergens at least several times/week for prevention [2]. Our case appears the first to highlight an extremely short period of deprivation leading to development of wheat allergy. Further research is needed to determine optimal frequency of continued ingestion of potential food allergens following first introduction, including whether more frequent ingestion is warranted for some allergens (e.g. wheat) compared to others (e.g. peanut), and the impact of age (infants vs. toddlers). Until more data is available, it would appear prudent to maintain regularity of any allergen at least once weekly.

We are confident that our patient and his family, given their experience with OIT and impressive changes in biomarkers, will be able to complete a home-based OIT regimen for his wheat allergy. However, given many other families lack experience with, time to commit to, or access to OIT, prevention should remain paramount in food allergy. Improved, unambiguous product labelling is one important way the food industry can support families with high-risk infants to ensure they are providing allergenic foods regularly.

## Data Availability

No datasets were generated or analysed during the current study.

## Abbreviations

OIT: Oral immunotherapy
SPT: Skin prick testing
